# Neural Working Memory Changes During a Spaceflight Analog With Elevated Carbon Dioxide: A Pilot Study

**DOI:** 10.3389/fnsys.2020.00048

**Published:** 2020-07-28

**Authors:** Ana Paula Salazar, Kathleen E. Hupfeld, Jessica K. Lee, Nichole E. Beltran, Igor S. Kofman, Yiri E. De Dios, Edwin Mulder, Jacob J. Bloomberg, Ajitkumar P. Mulavara, Rachael D. Seidler

**Affiliations:** ^1^Department of Applied Physiology and Kinesiology, University of Florida, Gainesville, FL, United States; ^2^Institute of Aerospace Medicine, German Aerospace Center, Cologne, Germany; ^3^KBR, Houston, TX, United States; ^4^NASA Johnson Space Center, Houston, TX, United States; ^5^Department of Neurology, University of Florida, Gainesville, FL, United States

**Keywords:** cognition, spatial working memory, carbon dioxide, head down tilt bed rest, microgravity

## Abstract

Spaceflight missions to the International Space Station (ISS) expose astronauts to microgravity, radiation, isolation, and elevated carbon dioxide (CO_2_), among other factors. Head down tilt bed rest (HDBR) is an Earth-based analog for spaceflight used to study body unloading, fluid shifts, and other factors unrelated to gravitational changes. While in space, astronauts need to use mental rotation strategies to facilitate their adaptation to the ISS environment. Therefore, spatial working memory is essential for crewmember performance. Although the effects of HDBR on spatial working memory have recently been studied, the results are still inconclusive. Here, we expand upon past work and examine the effects of HDBR with elevated CO_2_ (HDBR + CO_2_) on brain activation patterns during spatial working memory performance. In addition, we compare brain activation between 30 days of HDBR + CO_2_ and 70 days of HDBR to test the isolated effect of CO_2_. Eleven subjects (6 males, 5 females; mean age = 34 ± 8 years) underwent six functional magnetic resonance imaging (fMRI) sessions pre-, during, and post-HDBR + CO_2_. During the HDBR + CO_2_ intervention, we observed decreasing activation in the right middle frontal gyrus and left regions of the cerebellum, followed by post-intervention recovery. We detected several correlations between brain and behavioral slopes of change with the HDBR + CO_2_ intervention. For example, *greater* increases in activation in frontal, temporal and parietal regions were associated with *larger* spatial working memory improvements. Comparing the HDBR + CO_2_ group to data from our previous 70-day HDBR study, we found *greater decreases* in activation in the right hippocampus and left inferior temporal gyrus for the HDBR + CO_2_ group over the course of the intervention. Together, these findings increase our understanding of the neural mechanisms of HDBR, elevated levels of CO_2_ and spaceflight-related changes in spatial working memory performance.

## Graphical Abstract

Top: previous findings regarding spatial working memory performance after bed rest protocols. Citations are presented in the Reference section. Bottom: Summary of findings from the present study showing **(A)** no differences in spatial working memory behavior performance; **(B)** decreasing activation in some brain regions suggesting that HDBR + CO_2_ may reduce subjects’ ability to recruit appropriate working memory networks; **(C)** positive and negative brain-behavior correlations suggesting adaptive or compensatory brain response to HDBR + CO_2_.

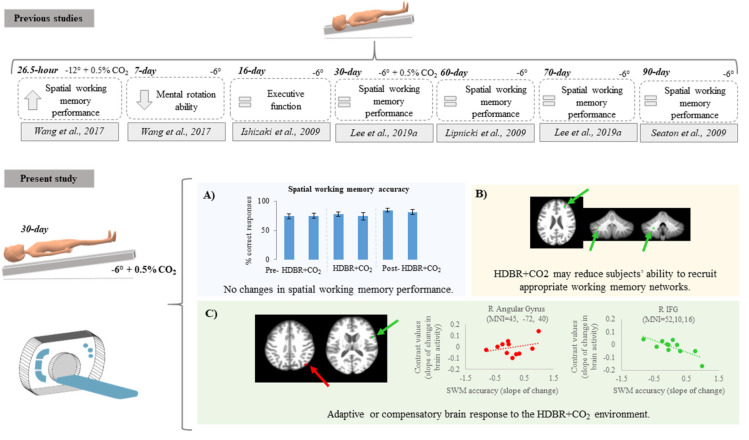

## Introduction

Spaceflight negatively affects human sensorimotor functioning and cognition (De la Torre, 2014). Cognitive performance in astronauts may be impaired by microgravity, radiation, noise, fatigue, and sleep deprivation, among other factors (De la Torre, 2014). Spatial orientation, mental rotation, and recognition are among the most common cognitive processes affected by spaceflight (De la Torre, 2014).

Mental rotation is a type of spatial working memory task in which a person imagines how an object would appear if it was rotated away from the presented orientation (Shepard and Metzler, 1971). Working memory is part of the short-term memory system, which involves a series of interactive processes that comprise the ability to temporarily maintain and manipulate information in the mind (Baddeley, 2017). Spatial working memory has an important role for executive function as well as sequence learning and sensorimotor adaptation (Seidler et al., 2012). Therefore, working memory is essential for successful crewmember performance. For instance, while in space, astronauts use mental rotation strategies to facilitate the recognition of objects and other astronauts’ gestures.

Head down tilt bed rest (HDBR) is a well-established Earth-based analog of spaceflight used to investigate the physiological effects of microgravity on human performance (Moore et al., 2010). HDBR simulates the axial body unloading and fluid shifts toward the head that occur during spaceflight. Both spaceflight and HDBR impact sensorimotor function and are associated with modifications of brain structure and function in healthy individuals (Bock et al., 2010; Koppelmans et al., 2016; Roberts et al., 2017; Lee et al., 2019b). The effects of HDBR specifically on working memory remain unclear. Previous work assessed 20 males that underwent seven days of −6° HDBR. These individuals showed *reduced* mental rotation ability after three days of HDBR, but recovered after the end of HDBR, suggesting that short-duration HDBR temporarily impacts mental rotation abilities (Wang et al., 2017). Our group previously evaluated 17 males who underwent a 70-day HDBR intervention. We reported *improvements* in spatial working memory performance after 70 days of HDBR compared to baseline, suggestive of test practice effects (Cassady et al., 2016). Further, we found that working memory performance changes correlated with brain connectivity alterations (Cassady et al., 2016). This suggests that neuroplastic mechanisms may facilitate adaptation to the HDBR environment (Cassady et al., 2016).

In addition to microgravity, chronic exposure to elevated levels of carbon dioxide (CO_2_) on the International Space Station (ISS) may also contribute to cognitive performance impairments (Manzey and Lorenz, 1998; Allen et al., 2019). Astronauts aboard the ISS often report hypercapnia-related symptoms such as headaches (Law et al., 2014), spatial disorientation, reduced attention and concentration, among other symptoms (Kanas and Manzey, 2008; De la Torre, 2014). Our group recently reported the effects of 30 days HDBR coupled with elevated CO_2_ on cognitive and sensorimotor performance (Lee et al., 2019a). Individuals in this cohort showed *improvements* in card rotation performance (i.e., a learning effect and no effect of the intervention) and *no changes* in cube rotation and working memory (Lee et al., 2019a). Although several recent studies have reported HDBR- and spaceflight-related changes in spatial working memory abilities (Leone et al., 1995; Lipnicki et al., 2009; Chen et al., 2013; Wang et al., 2017), there is still little understanding regarding how HDBR may affect the neural processing of spatial working memory. Further, no previous work has investigated neural spatial working memory changes with a combined HDBR and elevated CO_2_, which better mimics the elevated CO_2_ onboard the ISS (Law et al., 2014).

In the present pilot study, we examine the effects of 30 days of HDBR combined with elevated CO_2_ levels (HDBR + CO_2_) on the neural correlates of spatial working memory performance in eleven participants. We addressed two primary aims: (1) to investigate the time course of effects of a 30-day HDBR + CO_2_ intervention on brain activation patterns during spatial working memory task performance; and (2) to determine whether any brain changes correlate with changes in spatial working memory performance. As a secondary aim, to investigate the additive effects of elevated CO_2_ and long-duration HDBR, we compared the data here with those from our previous HDBR work (Yuan et al., 2016, 2018a; Koppelmans et al., 2017). This comparison was exploratory, given that the two HDBR interventions differed on several dimensions including the exposure duration.

## Materials and Methods

### Participants and Testing Timeline

#### HDBR + CO_2_

**FIGURE 1:**
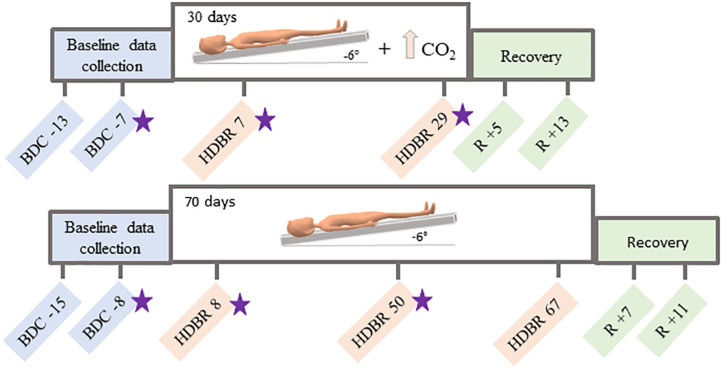
Testing timelines. *Top:* testing timeline for the HDBR + CO_2_ group, who completed 30 days of head down tilt bed rest (HDBR) with 0.5% atmospheric CO_2_. *Bottom:* testing timeline for the HDBR group, who completed 70 days of HDBR with normal atmospheric CO_2_ levels. BDC = baseline data collection; HDBR = head down tilt bed rest; R = recovery. Functional MRI and behavioral data were collected at all time points specified here. Stars indicate the three time points used to create the slope images for between-group comparisons.

This longitudinal study conducted at:envihab in the German Aerospace Center, Cologne, Germany, included eleven participants (6 males, 5 females) with mean age of 34 ± 8 years at the beginning of the study. Participants were tested in six different time points: twice before the protocol started, twice during intervention and twice after the end of the bed rest (Figure 1). During the HDBR + CO_2_ intervention, subjects maintained 6° head down tilt position while exposed to ambient 0.5% CO_2_ (3.8 mmHg partial pressure of CO_2_) (Law et al., 2014) at all times during 30 days. Oxygen and nitrogen levels were 20.9% and 78.6%, respectively. These small changes had no physiological effects neither affected the oxygen saturation. All participants received a controlled diet, had daily 8-h sleep opportunities (10:30 PM–6:30 AM) and were not allowed to use a pillow except when laying on their side.

Three days prior to bed rest and on the first day after bed rest blood draws were acquired to measure arterial partial pressure of carbon dioxide (PaCO_2_). This was part of NASA’s standard measures assessments.

All procedures were approved by the University of Florida and NASA Institutional Review Boards as well as by the local ethical commission of the regional medical association (Ärztekammer Nordrhein). All subjects provided written informed consent and received monetary compensation for their participation.

#### 70-day HDBR

Sixteen individuals (all males; mean age = 29 ± 3 years) consented to participate in this study. All procedures were approved by the University of Michigan, University of Texas Medical Branch, and NASA Institutional Review Boards. All participants were admitted to the NASA bed rest facility at the University of Texas Medical Branch, Galveston, TX, United States and completed two baseline data collection sessions in the 2 weeks prior to starting HDBR. Subjects then underwent 70 days of HDBR intervention with normal atmospheric CO_2_ (∼0.04%; 0.3 mmHg partial pressure of CO_2_). During this campaign, participants remained lying down with a six-degree head down tilt at all times. They were allowed to use a pillow and to support their head with their hand during each meal (30 min). Subjects stayed at the facility for 14 days after HDBR and completed two recovery data collection sessions during this time (Figure 1).

### Spatial Working Memory Behavioral Tasks

**FIGURE 2:**
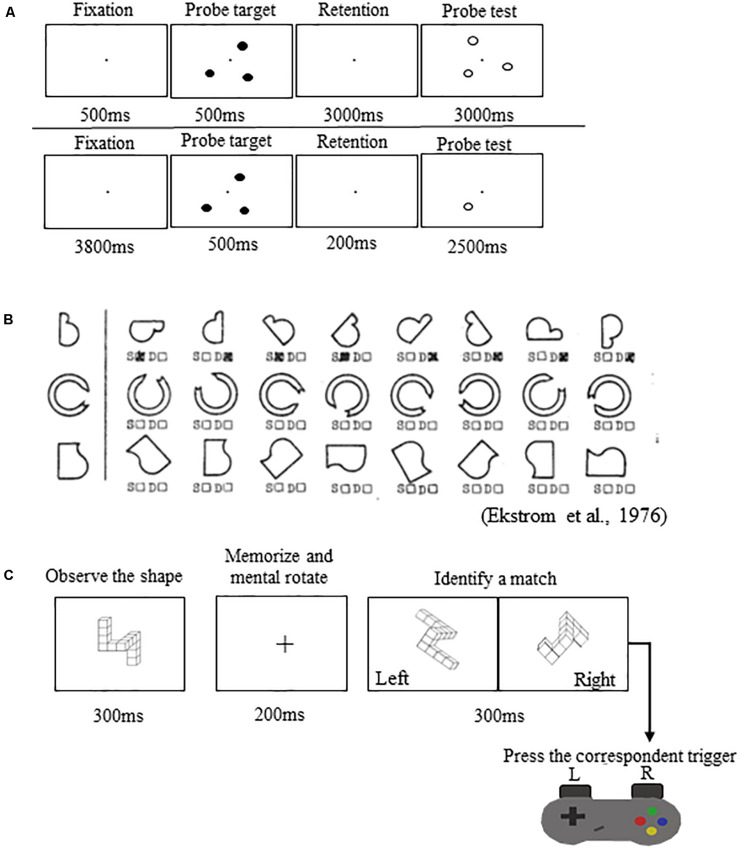
Spatial working memory performance tasks. **(A)** *Top*: Spatial working memory task performed in the MRI scanner. *Bottom*: Spatial working memory control task performed in the MRI scanner. **(B)** Thurstone’s 2D card rotation test. **(C)** Cube rotation task.

Spatial working memory behavioral tasks were acquired at all time points specified in Figure 1. Three different tasks were used to assess spatial working memory performance, as follows:

(1) *Spatial working memory task during functional magnetic resonance imaging (fMRI) (Figure 2A)*: This task was performed in the MRI scanner. Participants viewed a three-target set (three solid circles) for 500 ms. Following the presentation of this target set, participants saw a blank screen for 3000 ms (retention interval). During the retention interval, participants were instructed to mentally “connect the dots” and then mentally rotate the shape. After the retention interval, participants decided whether a subsequently presented probe set of open circles formed the same configuration as the target set they mentally rotated. Participants performed two runs of this task. Each run included 30 trials.

Participants also performed a control task in the MRI scanner (Figure 2A). The control task involved the presentation of three solid circles for 500 ms, followed by a 200 ms retention interval, then by the presentation of a single circle for 2500 ms. At this point, participants determined whether its spatial location matched that of a previously observed dot. Participants performed one run of this task consisting of 40 trials. This control task included all of the processes of the spatial working memory task, except for the working memory and mental rotation components. Thus, the subtraction of images from the control condition should reveal areas actively involved with spatial working memory maintenance and mental rotation while omitting those involved in visual processing and response button pressing (Reuter-Lorenz et al., 2000; Anguera et al., 2010). For both tasks, we calculated the percentage of correct responses (spatial working memory and spatial working memory control accuracy).

(2) *Card rotation (Figure 2B)*: Participants completed Thurstone’s 2D card rotation test (Ekstrom et al., 1976). During each trial, they were presented with a 2D drawing of a card with an abstract shape. To the right of this card, there were eight drawings of the same card that were either only rotated or both rotated and mirrored. Participants determined which cards matched the initial drawing (S = same, i.e., only 2D rotated) and which cards were different (D = different, i.e., mirrored or flipped from the card at the beginning of the row). Time to complete the test (maximum time allowed is 3 min) and accuracy relative to completed trials were used as indicators of performance (Koppelmans et al., 2013; Cassady et al., 2016).
(3) *Cube rotation (Figure 2C)*: Participants compared a collection of 3D cubes (Shepard and Metzler, 1988). During each trial, a 3D cube assemblage was presented on a computer screen for 3 s, followed by a blank screen for 2 s, and then two cube images. One of the two was a match to the target but was rotated three dimensionally; the other was a new cube assemblage. Participants indicated which cube image matched the target image by pressing a left or right button. Outcome measures for this task included reaction time and accuracy.

For both card and cube rotation assessments, the HDBR + CO_2_ participants were in head down tilt while subjects from 70-day HDBR performed this task in the supine position (Koppelmans et al., 2013; Cassady et al., 2016).

### fMRI Acquisition Parameters

#### HDBR + CO_2_

Functional images were acquired on a 3 Tesla Siemens MRI scanner, using a gradient echo T2^∗^-weighted echo-planar imaging sequence with the following parameters: TR = 2500 ms, TE = 32 ms, flip angle = 90°, FOV = 192 × 192 mm, matrix = 64 × 64, slice thickness = 3.5 mm, voxel size = 3 × 3 × 3.5 mm^3^, 37 slices. A T1-weighted gradient-echo pulse sequence was also acquired: TR = 1.9 s, TE = 2.4 ms, flip angle = 9°, FOV = 250 × 250 mm, matrix = 512 × 512, slice thickness = 1.0 mm, voxel size = 0.49 × 0.49 × 1.0 mm^3^, 192 slices. Participants maintained the head down tilt position in the scanner by lying on a wedge of foam; however, the head was supine in the head coil.

#### 70-day HDBR

For the 70-day HDBR group, fMRI scans were acquired on a 3 Tesla Siemens MRI scanner using a gradient echo T2^∗^-weighted echo-planar imaging sequence: Repetition time (TR) = 3.66 s, Echo time (TE) = 39 ms, flip angle = 90°, Field of view (FOV) = 240 × 240 mm, matrix = 94 × 94, slice thickness = 4 mm, slice gap = 1 mm, voxel size = 2.55 × 2.55 × 5.0 mm^3^, 36 slices. A T1-weighted gradient-echo pulse sequence was also collected with parameters: TR = 1.9 s, TE = 2.49 ms, flip angle = 9°, FOV = 270 × 270 mm, matrix = 288 × 288, slice thickness = 0.90 mm, voxel size = 0.94 × 0.94 × 0.90 mm^3^, 192 slices. Participants did not maintain the head down tilt position in the scanner.

### fMRI Data Processing and Statistical Analyses

We used Statistical Parametric Mapping 12 (SPM12, version 7219) and MATLAB R2018a, version 9.0 for preprocessing and statistical analyses. We used a standard SPM preprocessing pipeline for fMRI. All functional images were slice timing and head motion corrected (realigned and resliced). Following these steps, the Artifact Detection Tool (ART)^1^ was used as an additional quality check. We removed volumes with motion threshold equal or greater than 3 mm (i.e., approximately the size of one voxel for the HDBR + CO_2_ group) and global brain signal Z threshold equal or greater than 9. Two individuals had movement outliers; for one of them the first 21 of 76 volumes were excluded, while the first 8 of 76 volumes were excluded for the other participant. We included head motion parameters outputted by ART as covariates in the subject-level analyses to minimize effects of these volumes on group-level analyses.

Next, whole brain fMRI images were normalized to MNI152 space using Advanced Normalization Tools (Avants et al., 2011), in a multi-step procedure. First, the T1 images were skull stripped using ImCalc (SPM12). Then, participant-specific templates were created using ANTs’ *AntsMultivariateTemplateConstuction.sh* function. Next, these templates were normalized to MNI152 common space using ANTs’ *AntsRegistration.sh* function. In order to normalize the images, we then created mean fMRI participant-specific templates (using ANTs’ *AntsMultivariateTemplateConstuction.sh* function) and used these templates to coregister the functional images to the T1-specific templates. Coregistration was performed using *AntsRegistration.sh*. The resulting warp parameters were applied to the 4D EPI images using ANTs’ *AntsApplyTransforms.sh* function. Finally, the normalized data were spatially smoothed with an 8 mm full-width half-maximum three-dimensional Gaussian kernel.

In addition to the whole brain normalization, we applied specialized processing using portions of both the CEREbellum Segmentation (CERES) (Romero et al., 2017) pipeline and the Spatially Unbiased Infratentorial Template (SUIT) (Diedrichsen, 2006; Diedrichsen et al., 2009) pipeline. The CERES pipeline was used to segment the cerebellum from each person’s structural T1-weighted image. We then coregistered each subject’s native space segmentation to the *SUIT.nii* template. Binary gray matter, white matter, and full cerebellar masks were created from the CERES native space output, and we then used the *suit_normalize_dartel* function to obtain the affine transformation matrix and normalize these images into SUIT space. Due to the small size of cerebellar structures, we applied a 2 mm full-width half-maximum three-dimensional Gaussian smoothing kernel to the normalized functional cerebellar images.

We calculated subject-level statistical analyses twice: once for the whole brain and a second time for the cerebellum. Brain activity was calculated for each participant on a voxel-by-voxel basis for the contrast spatial working memory > spatial working memory control. We set the first level masking threshold to -Infinity and masked out non-brain areas using the SPM intracranial volume mask.

### fMRI Group-Level Statistical Analyses

#### Main Effect of Spatial Working Memory

To verify that our spatial working memory task elicited the expected brain activity, we calculated the main effect across all subjects and all sessions at peak-level of *p* < 0.001 (uncorrected), extent threshold = 10 voxels. In this model, we controlled for age and sex differences, i.e., these variables were included as covariates of no interest. For all analyses we used the contrast spatial working memory > control.

#### Time Course of Neural Spatial Working Memory Response to HDBR + CO_2_

**FIGURE 3:**
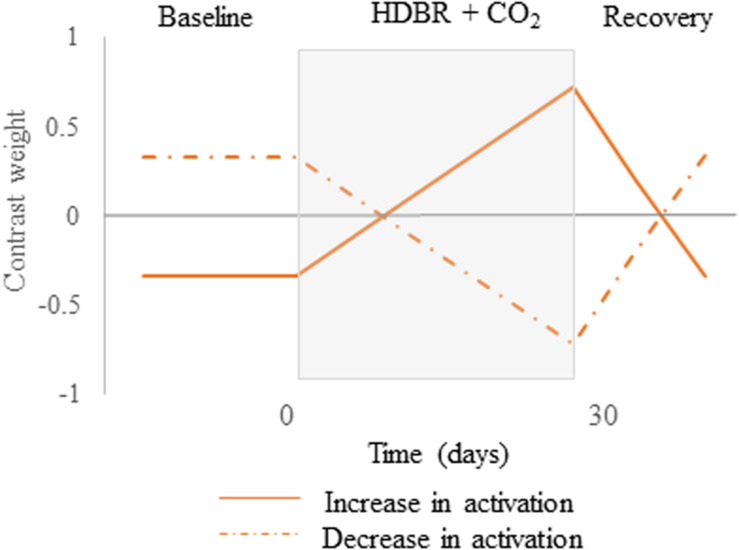
Hypothesized contrast weights. Cumulative changes in neural spatial working memory response to HDBR + CO_2_. Brain changes would slowly increase over the course of HDBR + CO_2_, followed by recovery after the end of HDBR + CO_2_. Solid lines represent the positive version while dotted lines describe the negative version of the contrast.

We first tested for brain regions that showed a pattern of cumulative change followed by post-HDBR + CO_2_ recovery. These hypothesized cumulative change models are presented in Figure 3. For these longitudinal analyses, we used flexible factorial models controlling for age and sex assuming independence between subjects, and assuming equal variances between and within subjects (Gläscher and Gitelman, 2008). To better detect within-subject changes with the longitudinal model used in the present pilot study, the alpha level was set at *p* < 0.0005 (uncorrected), and the extent threshold was set at 10 voxels for the whole brain and 5 voxels for the cerebellum.

#### Brain-Behavioral Correlations

First, we computed the slope of changes in brain activation (Yuan et al., 2016, 2018b; Hupfeld et al., 2020) between the 2nd, 3rd, and 4th time points (Figure 1). These are the time points immediately before HDBR started and during HDBR, respectively. Additionally, we computed the slope of changes in behavioral performance on the spatial working memory, card rotation, and cube rotation tasks across the same time points. We then correlated the slope of brain changes with the slope of changes in spatial working memory performance. For these analyses, we used the Statistical Non-Parametric Mapping (SnPM version 13)^2^ toolbox to run non-parametric permutation tests with 15,000 permutations, variance smoothing = 8 mm kernel for whole brain analyses and 2 mm kernel for cerebellar analyses, and controlling for age and sex. For these analyses, we used a non-parametric threshold of *p* < 0.0005 (uncorrected) and a minimum cluster size of 10 voxels for the whole brain and 5 voxels for the cerebellum.

#### HDBR + CO_2_ vs. 70-day HDBR Group Comparisons

Given that each of the two bed rest studies followed a different testing timeline, to examine differences in neural response between HDBR with and without elevated CO_2_, we compared only the slopes of change in brain activation between these two studies. We computed slopes of brain change for the 70-day HDBR group in an identical manner to those for the HDBR + CO_2_ group. Additionally, for each group, we computed intercept images (i.e., baseline brain activation during spatial working memory). We then normalized the slope images using the formula: (slope image/intercept image); this allows us to compare between-group slope changes while accounting for baseline differences between the two groups.

We performed a two-sample *t*-test to test between-group differences in the normalized slope images. We used SnPM non-parametric permutation tests with 15,000 permutations, variance smoothing = 8 mm kernel for the whole brain analyses and 2 mm kernel for the cerebellar analyses, and controlling for age and sex. Statistical significance was determined by applying false discovery rate (FDR) *p* < 0.05 at the cluster-level (Nichols and Hayasaka, 2003).

### Statistical Analyses

A paired sample one tailed *t*-test was performed to verify any increases pre- to post-HDBR + CO_2_ in the PaCO_2_ blood levels. Statistical significance was defined as *p* < 0.05.

We previously reported some statistical analyses of the spatial working memory behavioral data for the HDBR + CO_2_ cohort (Lee et al., 2019a). Here, we further investigated the spatial working memory score using the following equation: Spatial working memory Score = Spatial working memory control accuracy – Spatial working memory accuracy. We did not have any outliers nor missing data. We conducted a linear mixed model regression analysis on the HDBR + CO_2_ participants, entering time as a continuous variable to assess the effect of the intervention on spatial working memory score. We used R software version 3.6.0 for this analysis entering time as a continuous variable, and age and sex as covariates. We considered the first time point to be a practice session and thus excluded it from the analysis (Lee et al., 2019a).

## Results

We observed a small but significant increase in PaCO_2_ from pre- (41.4 mmHg) to post- (43.4 mmHg) bed rest (*p* < 0.05).

### Spatial Working Memory Behavioral Results

We did not observe an effect of HDBR + CO_2_ on spatial working memory accuracy score (β = 0.12; *p* = 0.39). We previously reported the effects of HDBR + CO_2_ on spatial working memory (β = −0.03; *p* = 0.76), spatial working memory control (β = 0.10; *p* = 0.18), card rotation (time: β = −0.30; *p* < 0.01; accuracy: β = 0.11; *p* < 0.05), and cube rotation (time: β = −0.01; *p* = 0.18 and accuracy: β = −0.15; *p* = 0.15) (Lee et al., 2019a). We only found effects of time on card rotation time and accuracy, in which subjects showed improvement in both measures across HDBR + CO_2_ (Lee et al., 2019a).

### Main Effect of Spatial Working Memory

**FIGURE 4:**
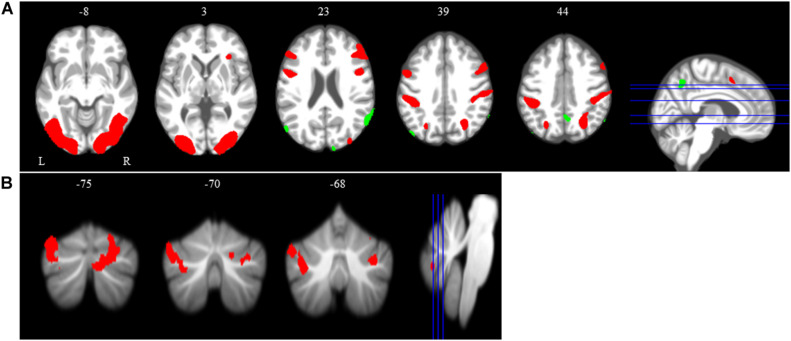
Main effect of spatial working memory. The spatial working memory task resulted in widespread activation (red) and deactivation (green). Whole brain and cerebellar results are overlaid onto MNI **(A)** and SUIT **(B)** standard templates, respectively; *p* < 0.001, *k* = 10. Abbreviations: L = Left; R = Right.

The main effect of the spatial working memory task contrasted to the control task resulted in activation in the expected brain regions based on prior studies (Lamp et al., 2016). Specifically, we observed bilateral activation in several frontal, parietal, temporal and cerebellar regions (Table 1 and Figure 4). We also found deactivation in parietal and occipital regions (Table 1 and Figure 4).

**TABLE 1:** Brain regions showing activation or deactivation during spatial working memory.

	Extent (*k*)	Peak t-value	MNI coordinates (mm)		
			*x*	*y*	*z*
**Activation**					
*Frontal*					
R IFG (p. Opercularis)	3989	5.301	50	7	30
R IFG (p. Triangularis)	4049	4.884	44	30	20
L IFG (p. Opercularis)	4296	5.734	−52	8	32
L IFG (p. Triangularis)	1428	4.026	−44	30	20
L Posterior-medial frontal	1714	4.771	−6	16	51
*Temporal*					
R Fusiform gyrus	28771	8.009	34	−81	−8
R Middle occipital gyrus	28771	7.308	25	−92	10
*Parietal*					
R Superior parietal lobule	13689	6.279	27	−58	53
R Post-central gyrus	13689	5.031	54	−22	40
L Inferior parietal lobule	9058	5.087	−30	−56	59
L Post-central gyrus	9058	4.886	−43	−38	51
*Occipital*					
R Middle occipital gyrus	28771	7.308	25	−92	10
L Lingual gyrus	21522	8.799	−18	−92	−7
L Inferior occipital gyrus	21522	6.725	−43	−72	−9
*Cerebellum*					
L Cerebelum (Crus 1)	21522	3.333	−10	−77	−23
L Cerebelum (VIII)	167	3.890	−16	−68	−47
**Deactivation**					
*Temporal*					
R Middle temporal gyrus	2188	–5.441	58	−59	23
L Angular gyrus	1094	–4.144	−44	−76	39
L Middle temporal gyrus	1094	–3.486	−47	−56	16
*Parietal*					
R Precuneus	832	–3.705	3	−54	47
R Inferior parietal lobule	21	–3.577	56	−59	44
*Occipital*					
R Cuneus	326	–4.642	12	−96	20
L Superior occipital gyrus	93	–3.818	−23	−92	30
L Superior occipital gyrus	23	–3.553	−11	−104	15

*Significance level set at non-parametric *p* < 0.001 and cluster size *k* = 10 for all analyses. Cortical regions labeled using the AnatomyToolbox atlas via the SPM toolbox BSPMview. Cerebellar regions labeled using the SUIT atlas. L = Left; R = Right.*

### Time Course of Neural Working Memory Response to HDBR + CO_2_

**FIGURE 5:**
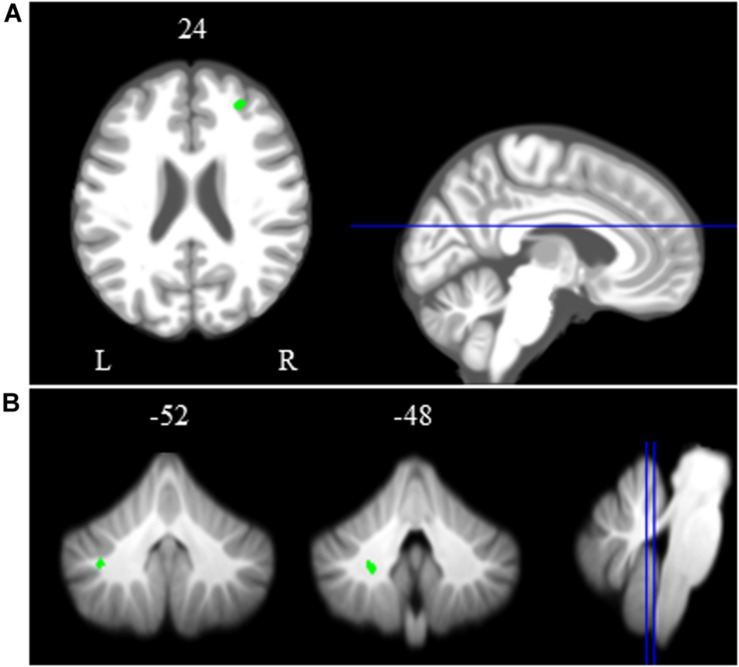
Time course of neural spatial working memory response to HDBR + CO_2_. Whole brain and cerebellar results showing decreases in activation (green) followed by recovery. Whole brain and cerebellar results are overlaid onto MNI **(A)** and SUIT **(B)** standard templates, respectively; *p* < 0.0005, *k* = 10 for whole brain analyses; *k* = 5 for cerebellar analyses. Abbreviations: L = Left; R = Right.

Across HDBR + CO_2_, we found *decreasing* activation in the right middle frontal gyrus and left dentate nucleus of the cerebellum, followed by recovery after the HDBR + CO_2_ intervention (Figure 5 and Table 2). We did not observe any increases in brain activation followed by recovery in response to HDBR + CO_2_.

**TABLE 2:** Brain regions showing cumulative changes during spatial working memory followed by recovery.

	Extent (*k*)	Peak *t*-value	MNI coordinates (mm)		
			*x*	*y*	*z*
**Decreases in activation**					
*Frontal*					
R Middle frontal gyrus	62	−3.969	28	44	24
*Cerebellum*					
L Dentate	8	−4.175	−22	−48	−41

*Significance level set at non-parametric *p* < 0.0005 and cluster size *k* = 10 for the whole brain analyses and *k* = 5 for the cerebellum analyses. Cortical regions labeled using the AnatomyToolbox atlas via the SPM toolbox BSPMview. Cerebellar regions labeled using the SUIT atlas. L = Left; R = Right.*

### Brain-Behavior Correlations

#### Spatial Working Memory Task

**FIGURE 6:**
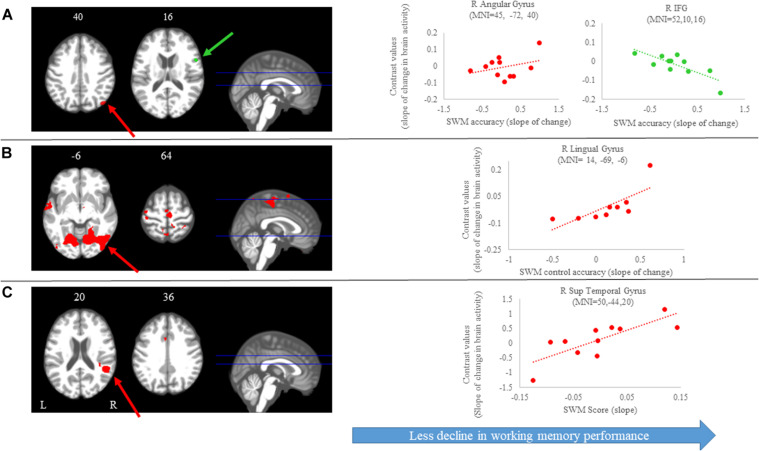
Brain-behavior correlations (spatial working memory). Slope of changes in brain and behavior results showing positive (red) and negative (green) correlations. **(A)** Spatial working memory; **(B)** spatial working memory control; and **(C)** spatial working memory score. Whole brain results are overlaid onto the MNI standard template; *p* < 0.0005, *k* = 10. Right side correlation plots include contrast values extracted from the peak coordinate inside an example cluster (indicated with red or green arrows) graphed against the slope of changes in behavior results. Abbreviations: L = Left; R = Right; SWM = Spatial working memory.

We identified several regions for which the slope of change in brain activity correlated with the slope of change in spatial working memory performance (Figure 6A and Table 3). For instance, for spatial working memory accuracy, we observed that *greater increases* in activation of the right angular gyrus were associated with *larger improvements* in spatial working memory performance. That is, subjects who performed this task with fewer errors presented with *greater increases* in activation of the right angular gyrus during the HDBR + CO_2_ intervention. Further, a *greater decrease* in activation of the inferior frontal gyrus was correlated with *less decline* in spatial working memory accuracy. For the spatial working memory control task, we found that *greater increases* in activation of several brain regions, including parietal, temporal and occipital regions, correlated with *greater* accuracy increases. In addition, *greater decrease* in activation of the left lingual gyrus was correlated with *less decline* in the accuracy of the spatial working memory control task (Figure 6B and Table 3). Regarding the spatial working memory scores, we observed that *greater increases* in activation of the right superior temporal gyrus were correlated with *greater increases* in scores (Figure 6C and Table 3).

**TABLE 3:** Brain regions showing associations between the slopes of change in brain and behavioral during spatial working memory.

	Extent (*k*)	Peak *t*-value	MNI coordinates (mm)		
			*x*	*y*	*z*
**Spatial working memory task**					
**Positive correlation**					
*Parietal*					
R Angular gyrus	36	4.3964	45	−72	40
**Negative correlation**					
*Frontal*					
R IFG (p. Opercularis)	12	5.2687	52	10	16
**Spatial working memory control**					
**Positive correlation**					
*Frontal*					
R Posterior-medial frontal	62	5.135	2	8	70
R Superior frontal gyrus	72	5.249	25	64	16
R IFG (p. Opercularis)	42	4.693	62	16	18
R Posterior-medial frontal	49	5.104	2	10	71
R Middle frontal gyrus	10	3.881	26	22	42
L Precentral gyrus	435	7.229	−40	−24	64
*Insula*					
L Insula lobe	74	4.626	−33	−29	22
*Temporal*					
R Superior temporal gyrus	182	6.253	48	−40	12
R Superior temporal gyrus	151	5.498	64	−12	12
R Superior temporal gyrus	89	6.537	53	−30	14
R Inferior temporal gyrus	248	6.552	46	−48	−16
R Medial temporal pole	256	8.568	34	14	−32
R Medial temporal pole	135	6.058	54	10	−20
R Medial temporal pole	17	5.584	60	6	−16
R Inferior temporal gyrus	77	3.341	43	−9	−34
R Temporal pole	32	3.049	48	5	−17
R Inferior temporal gyrus	10	3.508	58	−56	−20
L Middle temporal gyrus	40	5.870	44	−70	18
L Superior temporal gyrus	2192	12.836	−50	−16	10
L Superior temporal gyrus	2192	8.276	−50	−38	20
L Superior temporal gyrus	346	6.671	−56	0	−2
L Inferior temporal gyrus	96	3.870	−42	−42	−12
L Temporal pole	62	5.487	−32	10	−30
*Parietal*					
R Postcentral gyrus	796	5.512	26	−44	70
R Postcentral gyrus	151	7.760	60	−14	32
R Precuneus	91	4.977	2	−54	64
R Precuneus	45	3.772	4	−58	44
L Postcentral gyrus	2192	4.568	−58	−16	34
L Postcentral gyrus	435	10.650	−22	−34	78
*Occipital*					
R Lingual gyrus	8102	8.120	14	−69	−6
R Inferior occipital gyrus	3295	7.405	44	−68	−8
R Calcarine gyrus	966	4.887	24	−59	14
R Fusiform gyrus	41	5.093	40	−26	−30
R Fusiform gyrus	29	3.908	30	−6	−34
R Superior occipital gyrus	3295	7.760	18	−86	34
L Calcarine gyrus	155	7.554	−16	−100	0
L Fusiform gyrus	1482	5.699	−30	−76	−8
L Lingual gyrus	3295	8.011	14	−68	−6
L Superior occipital gyrus	40	6.671	−56	0	−2
L Inferior occipital gyrus	180	5.789	−43	−75	2
L Inferior occipital gyrus	70	5.356	−21	−101	−1
L Middle occipital gyrus	1482	7.826	−46	−70	4
*Subcortical*					
R Pallidum	38	3.691	24	−12	6
**Negative correlation**					
*Occipital*					
L Lingual gyrus	27	4.8595	−16	−102	−10
**Spatial working memory Score**					
**Positive correlation**					
*Frontal*					
R IFG (p. Opercularis)	15	3.8465	58	16	16
R Posterior-medial frontal	18	3.1255	−10	−14	58
L ACC	43	4.6595	−2	14	36
*Temporal*					
R Superior temporal gyrus	486	7.0509	50	−44	20
L Superior temporal gyrus	14	3.8465	−48	−8	2
*Occipital*					
R Fusiform gyrus	34	4.1826	28	−4	−38
**Card rotation (accuracy)**					
**Positive correlation**					
*Parietal*					
L Supramarginal gyrus	17	3.560	−62	−38	32
**Negative correlation**					
*Frontal*					
L Superior frontal gyrus	57	5.441	−20	−10	78
*Parietal*					
R Angular gyrus	42	4.006	42	−66	38
*Occipital*					
L Lingual gyrus	30	4.238	−32	−88	−10
**Cube rotation (time)**					
**Positive correlation**					
*Parietal*					
L Postcentral gyrus	47	3.2271	−18	−46	48
*Occipital*					
R Middle occipital gyrus	41	4.7345	32	−88	16
L Fusiform gyrus	29	4.2604	−24	−44	−10
**Cube rotation (accuracy)**					
**Positive correlation**					
*Frontal*					
R Precentral gyrus	96	4.600	48	−8	56
R Precentral gyrus	18	3.167	54	4	40
R Superior frontal gyrus	37	3.669	22	34	50
R Mid orbital gyrus	40	4.238	8	36	−6
*Temporal*					
R Middle temporal gyrus	68	4.307	64	−8	−12
R Middle temporal gyrus	24	3.516	52	−62	16
R ParaHippocampal gyrus	24	2.754	30	−4	−24
R Fusiform gyrus	12	2.524	45	−36	−19
R Inferior temporal gyrus	16	4.576	54	−6	−34
L Middle temporal gyrus	71	4.378	−60	−10	−10
L Hippocampus	91	4.493	−20	−32	0
L Middle temporal gyrus	33	3.517	−50	−68	12
L Middle temporal gyrus	104	3.232	−64	−32	0
L Inferior temporal gyrus	17	3.232	−56	−16	−28
L Fusiform gyrus	15	2.821	−33	−36	−23
*Parietal*					
R Postcentral gyrus	15	3.028	62	−9	39
R Postcentral gyrus	15	2.366	63	−6	38
L Angular gyrus	30	3.588	−42	−74	42
*Subcortical*					
L Thalamus	29	2.783	−16	−23	14
L Pallidum	13	2.149	−22	−6	3

*Significance level set at non-parametric *p* < 0.0005 and cluster size *k* = 10 for all analyses. Brain regions labeled using the AnatomyToolbox atlas via the SPM toolbox BSPMview. L = Left; R = Right; IFG = Inferior Frontal Gyrus; ACC = Anterior Cingulate Cortex.*

#### Card Rotation Task

**FIGURE 7:**
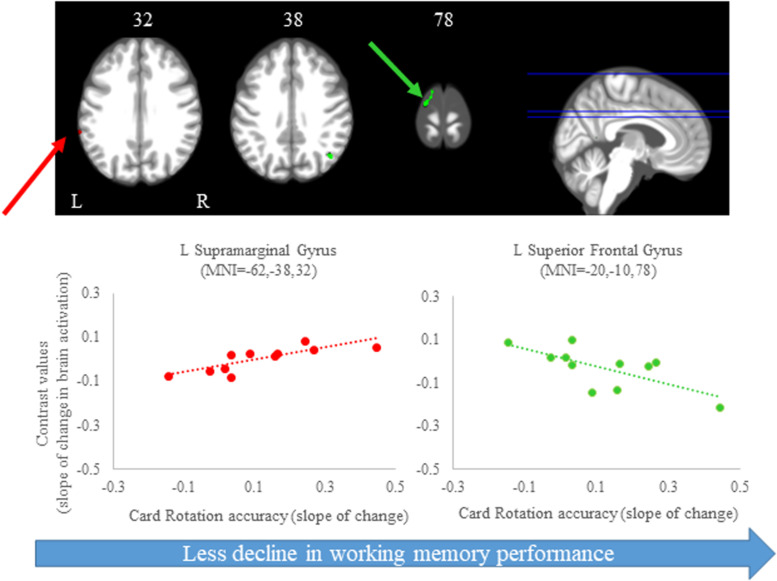
Brain-behavior correlations (card rotation task). Slope of changes in brain and behavior results showing positive (red) and negative (green) correlations. *Top:* Whole brain results overlaid onto the MNI standard template; *p* < 0.0005, *k* = 10. *Bottom:* Correlation plots include contrast values extracted from the peak coordinate inside an example cluster (indicated with red or green arrows) graphed against the slope of changes in behavior results. Abbreviations: L = Left; R = Right.

We observed several brain regions for which the slope of change in brain activity correlated with the slope of change in card rotation accuracy (Figure 7 and Table 3). We found that *greater increases* in activation of the left supramarginal gyrus correlated with *larger improvements* in card rotation accuracy, while *greater decreases* in activation of the left superior frontal gyrus, right angular gyrus, and left lingual gyrus correlated with *less decline* in this measure (Figure 7 and Table 3). We did not observe brain and behavior correlation for the time to perform the card rotation task.

#### Cube Rotation Task

**FIGURE 8:**
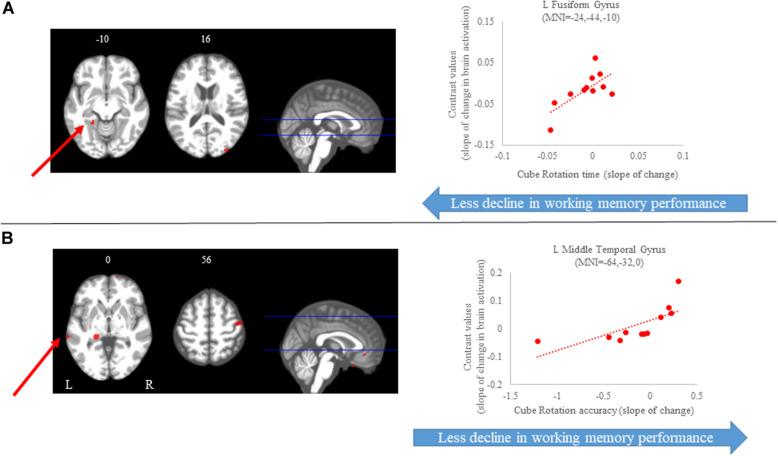
Brain-behavior correlations (cube rotation task). Slope of changes in brain and behavior results showing positive (red) correlations. **(A)** Cube rotation time; and **(B)** cube rotation accuracy. Whole brain results are overlaid onto the MNI standard template; *p* < 0.0005, *k* = 10. Right side correlation plots include contrast values extracted from the peak coordinate inside an example cluster (indicated with red arrows) graphed against the slope of changes in behavior results. Abbreviations: L = Left; R = Right.

For the cube rotation task, we observed several regions in which the slope of change in brain activity correlated with the slope of change in cube rotation accuracy. We found that *greater increases* in activation of several brain regions, including frontal, parietal, temporal and subcortical regions, were correlated with *greater* accuracy increases. That is, participants who *increased activation* in these brain regions presented with *better* accuracy on this task. For the time to perform the cube rotation task, we found that those subjects who showed *increases activation* of the left postcentral gyrus, left fusiform gyrus, and right middle occipital gyrus required *less time* to perform the task (Figure 8 and Table 3).

### HDBR + CO_2_ vs. 70-day HDBR Group Comparisons

Between-group normalized slope comparisons for HDBR + CO_2_ and 70-day HDBR revealed differences in the right hippocampus and left inferior temporal gyrus (Table 4). That is, the HDBR + CO_2_ group showed *greater decreases* in activation in both brain regions across the intervention in comparison to HDBR alone. These results were detected at the conservative, corrected statistical threshold of FDR < 0.05 (Nichols and Hayasaka, 2003).

**TABLE 4:** Regions with differences in slope of change in brain activation during bed rest between HDBR + CO_2_ and HDBR.

	Extent (*k*)	Peak *t*-value	MNI coordinates (mm)		
			*x*	*y*	*z*
**HDBR + CO_2_ < HDBR**					
*Temporal*					
R Hippocampus	12	3.444	18	−40	14
L Inferior temporal gyrus	11	3.586	−66	−44	−14

*Significance level set at FDR *p* < 0.05 and cluster size *k* = 10. Brain regions labeled using the AnatomyToolbox atlas via the SPM toolbox BSPMview. L = Left; R = Right.*

## Discussion

This is the first study to investigate the effects of 30 days of HDBR combined with elevated CO_2_ on brain activation during spatial working memory performance. While there were no group-level declines in spatial working memory performance, we observed *decreases* in brain activation in several cortical and cerebellar regions in response to the HDBR + CO_2_ intervention, followed by recovery. In addition, we found that, in general, individuals who exhibited *greater increases* in brain activation also showed *less declines* in spatial working memory performance. The right superior temporal gyrus showed differential changes between the HDBR + CO_2_ and 70-day HDBR groups, suggesting that elevated CO_2_ levels may particularly affect the function of this brain region.

### Spatial Working Memory Behavioral Results

Here, we found no differences in spatial working memory accuracy and score (which compares accuracy of the working memory condition to that of the control condition) or cube rotation time and accuracy across 30-days of HDBR + CO_2_. Likewise, Ishizaki et al. (2009) evaluated the effects of a 16-day HDBR intervention on executive function in young healthy participants and found no intervention-related changes in performance. Seaton et al. (2009) also found no differences in cognition after 60 or 90 days of HDBR. In contrast, Wang et al. (2017) assessed male healthy subjects that underwent 7 days of HDBR and reported poorer mental rotation accuracy in comparison to baseline. Similarly, Lipnicki et al. (2009) studied the effects of 60-day HDBR and found declines in working memory performance. Another study also examined healthy young individuals regarding time-based prospective memory with an ongoing word recall task and identified impaired prospective memory during HDBR compared to baseline (Chen et al., 2013). Although the behavioral effects of HDBR on spatial working memory are mixed, it seems that long-duration HDBR largely does *not* affect working memory abilities, with only one study showing differences due to 7 days of HDBR.

### Time Course of Spatial Working Memory Response to HDBR + CO_2_

We identified decreasing activation in the right middle frontal gyrus and the cerebellar dentate nucleus, followed by recovery. These brain regions are involved in attention, mental rotation, and reorientation and are commonly activated during spatial working memory tasks (Thürling et al., 2012; Japee et al., 2015). Thus, these longitudinal changes suggest that HDBR + CO_2_ may have reduced the subjects’ ability to recruit appropriate working memory networks, or alternatively that it increased neural efficiency. We previously reported that the upward shift of the brain with both spaceflight (Koppelmans et al., 2016) and bed rest (Koppelmans et al., 2017) results in apparent reductions in gray matter volume of this region, which could potentially reflect gray matter compression. These structural brain changes may relate to the reduction in activation of this region during spatial working memory performance in the current study.

### Brain-Behavior Correlations

We observed multiple brain-behavior correlations for the spatial working memory, 2D card rotation and 3D cube rotation tasks. Thus, although card rotation time and accuracy were the only behavioral metrics that changed with the intervention (Lee et al., 2019a), individual differences in performance changes in all tasks associated with individual differences in brain activity changes. We found that *greater increases* in activation in parietal, temporal, and occipital brain regions were correlated with *larger improvements* in spatial working memory accuracy. These associations may represent an adaptive or compensatory brain response to the HDBR + CO_2_ environment. In the past, our group has demonstrated associations between changes in spatial working memory performance (assessed by cube and card rotation tasks) and changes in brain connectivity between sensorimotor seed regions and brain areas associated with spatial cognition after 70 days of HDBR (Cassady et al., 2016). Those participants who had the *greatest improvements* in spatial working memory performance showed the *greatest changes* in connectivity between the seed and target brain areas. Thus, in the present study, it could be that these brain-behavior associations represent an adaptive neural response and are related to HDBR more generally and not specifically to elevated levels of CO_2_.

### HDBR + CO_2_ vs. 70-day HDBR Group Comparisons

As we did not observe group differences in spatial working memory behavioral performance between the HDBR + CO_2_ and 70-day HDBR groups (Lee et al., 2019a), we expected to find few between-group differences in brain activation. We found that the HDBR + CO_2_ group presented a steeper slope of change in brain activity in several brain regions. That is, participants who underwent 30 days of HDBR + CO_2_ had *greater decreases* in activation in the right hippocampus and left inferior temporal gyrus than in HDBR alone. This was the only effect we observed that survived correction for multiple comparisons (FDR < 0.05). Previous functional neuroimaging studies have suggested that the inferior temporal gyrus is involved in several cognitive processes such as visual memory storage and cognitive learning (Miyashita, 1993). In the present study, *greater increases* in activation in the left inferior temporal gyrus was also correlated with *larger* improvements in cube rotation accuracy, which suggests compensatory network engagement to maintain performance during the intervention.

Similarly, the hippocampus plays an important role in long-term memory and working memory processing (Ni et al., 2017). Toepper et al. (2010) found activation in the right hippocampus when participants engaged in a spatial working memory task (Toepper et al., 2010). A more recent study with polar expeditioners who spent 14 months at the German Neumayer III station in Antarctica–a spaceflight analog model to study the effects of social isolation and environmental deprivation–observed reduced hippocampal volume in several regions following the expedition (Stahn et al., 2019). They also reported that reduced hippocampal volume was not associated with general cognitive performance, but it was correlated with performance on a spatial mental rotation task. Thus, it is possible that the hippocampal activation changes we observed here during the mental rotation working memory task occurred at least partly as a result of our subjects being isolated for 30 days.

Another recent study evaluated the effect of acute exposure to elevated levels of CO_2_ (0.5%) during HDBR in comparison to HDBR alone on cognitive performance. They reported that subjects exposed to 26.5 h of 12° HDBR + CO_2_ presented with greater accuracy and lower speed on the Visual Object Learning Task in comparison to HDBR alone (Basner et al., 2018). Based on that finding, Scully et al., speculated that the medial temporal cortex and the hippocampus could be more sensitive to changes in CO_2_ concentration, with concomitant improvement in memory performance (Scully et al., 2019). Our results are in agreement with their speculation since we observed that the elevated CO_2_ levels combined with HDBR had a small effect on these same brain regions. However, we did not observe significant improvements in working memory performance; it could be that our task was not sensitive enough to detect subtle CO_2_-induced changes in working memory performance. In combination, these studies support that medial temporal lobe and hippocampal changes with HDBR + CO_2_ could be due to some combination of HDBR, CO_2_, and/or isolation.

CO_2_ has a vasodilation effect which results in increased brain blood flow (Atkinson et al., 1990; Zhou et al., 2008) and consequently increased intensity of the blood oxygen level-dependent (BOLD) signal measured by fMRI (Corfield et al., 2001). However, the effects of elevated CO_2_ on brain perfusion are still inconclusive. In the present study, even though participants presented increases in their PaCO_2_ levels from pre- to post-HDBR + CO_2_ we did not see increases in brain activity in comparison to HDBR alone. Conversely, HDBR + CO2 presented greater decreases in activation in comparison to HDBR alone. On the other hand, a recent study from our group (again using the same subjects as in the present study) showed *greater increases* in activation of several regions during vestibular stimulation for the HDBR + CO_2_ group in comparison to the 70-day HDBR group (Hupfeld et al., 2020). This result suggests interactive or additive effects of bed rest and elevated CO_2_ for vestibular changes (Hupfeld et al., 2020), but not for spatial working memory changes. Therefore, again, elevated CO_2_ effects seem to be task-specific rather than global effects of HDBR or CO_2_.

### Limitations

This study has several limitations. First, we had a small sample size and thus the results should be generalized with caution. Second, the testing timelines differed between the HDBR + CO_2_ and 70-day HDBR groups; each group was part of a separate bed rest campaign. These data were collected on two different Siemens scanners with two slightly different fMRI sequences. The HDBR + CO_2_ fMRI sequence included a faster TR than the 70-day HDBR sequence. However, we controlled for these differences as much as possible by using age and sex as covariates and by using slope comparisons to account for timeline differences (Yuan et al., 2016, 2018b; Hupfeld et al., 2020). Third, although the between-group comparison is FDR corrected (i.e., a more conservative statistical threshold), due to the limited pilot sample size, we used uncorrected *p*-values for the other neuroimaging statistical analyses to better detect within- and between-subject differences (Hupfeld et al., 2020). It is known that there is an upward shift of the brain and fluid redistribution during HDBR (Koppelmans et al., 2017), so it is not clear whether or how those changes interact with the functional brain changes seeing here. The fourth caveat of this study is that subjects on the HDBR + CO_2_ group underwent stricter bed rest, so it is unclear whether the results found here are due to the effects of the elevated levels of CO_2_ and/or the absence of a pillow in certain postures. Additionally, subjects in the HDBR campaign were scanned while supine, whereas those in the HDBR + CO_2_ were maintained at −6°. Finally, it should also be mentioned that HDBR + CO_2_ mimics only some of the effects of spaceflight, such as high levels of CO_2_, body unloading and fluid shifts toward the head, so it is difficult to fully generalize these findings to spaceflight. Moreover, lung volumes are reduced in bed rest and microgravity but not with the same extent (West, 2000; Prisk, 2005), then elevated CO_2_ levels may have larger effects on the ISS than on Earth.

## Conclusion

We investigated the longitudinal neural effects of HDBR + CO_2_ on spatial working memory. We observed decreases in activation in brain regions that are involved in attention, mental rotation and reorientation followed by recovery. This suggests that 30 days of HDBR combined with elevated CO_2_ levels may reduce the ability to recruit these brain regions. These findings contribute to a better understanding of how the working memory system adapts to a spaceflight analog environment.

## Data Availability Statement

The raw data supporting the conclusions of this article will be made available by the authors, without undue reservation.

## Ethics Statement

The studies involving human participants were reviewed and approved by the University of Florida Institutional Review Board. NASA Institutional Review Board. Local ethical commission of the regional medical association (Ärztekammer Nordrhein). The patients/participants provided their written informed consent to participate in this study.

## Author Contributions

AS analyzed the spatial working memory fMRI and behavioral data, created the figures and tables, and wrote the first draft of the manuscript. KH assisted with fMRI preprocessing, fMRI statistical analyses, and preparation of the initial manuscript draft. JL and EM collected, analyzed, and managed the data. NB and YD collected and analyzed the data. IK participated in project design and software development. JB, AM, and RS designed the project, secured funding, and led the interpretation and discussion of the results. All authors participated in revision of the manuscript.

## Conflict of Interest

NB, IK, YD, and AM were employed by the company KBR. The remaining authors declare that the research was conducted in the absence of any commercial or financial relationships that could be construed as a potential conflict of interest.

## Footnotes

1 www.nitrc.org/projects/artifact_detect/

2 http://warwick.ac.uk/snpm
